# Effects of dietary iron sources on growth performance, iron status, Fe-containing enzyme activity and gene expression related to iron homeostasis in tissues of weaned pigs

**DOI:** 10.3389/fvets.2023.1111257

**Published:** 2023-03-08

**Authors:** Ru-Qu Huang, Xiao-Jie Yang, Gao-Mao Xie, Jie Li, Yun-Hua Jian, Jing Yang, Yong-Wen Zhu

**Affiliations:** ^1^Guangdong Guangken Animal Husbandry Group Co., Ltd., Guangzhou, China; ^2^Guangdong Provincial Key Laboratory of Animal Nutrition and Regulation, College of Animal Science, South China Agricultural University, Guangzhou, China; ^3^Guangdong AIB Polytechnic College, Guangzhou, China

**Keywords:** weaned pig, iron sources, growth performance, iron status, gene expression

## Abstract

The aim of this study is to evaluate the effects of dietary iron sources on growth performance, iron status and activities of Fe-containing enzymes and gene expression related to iron homeostasis in tissues of weaned pigs. A total of 480 piglets at d 28 (Duroc X Landrace) were allotted to four groups as a factorial arrangement of treatments with 30 pigs/pen (male: female = 1:1) and 4 replicate pens/treatment. The treatments for iron in the diets were: control basal diet (Con); Con + 150 mg Fe/kg as inorganic Fe (iFe); Con + 75 mg Fe/kg as inorganic Fe + 75 mg Fe/kg as organic Fe-peptide complex (iFe+oFe) and Con + 150 mg of Fe/kg as organic Fe-peptide complex (oFe). The feeding trial lasted for 36 days. There were no significant differences in final body weight, ADG, ADFI, and G/F as well as blood hemoglobin and MCHC contents between piglets fed the control and iron-supplemented groups (*P* > 0.05). The iron supplemented groups exhibited increased iron content in the liver, kidney and spleen as well as the CAT and SDH activities in liver compared to the control group (*P* < 0.05), while piglets in oFe group experienced greater Fe accumulation and activities of CAT and SDH in the liver than piglets in the iFe group. Compared with the control group, dietary supplementation of iron increased the *NCOA4* mRNA expression and decreased the *TfR1* mRNA expression in liver of piglets. The *TfR1, NCOA4* and *Ferritin* mRNA expressions of bone marrow in both iFe and iFe+oFe groups were greater than both in the Con and oFe groups. These results suggest that dietary supplementation of iron does not influence the growth performance and hematological parameters in weaned pigs fed a corn-soybean meal basal diet (75.8 mg/kg) from d 28 to d 70, but increased tissue iron status and activities of Fe-containing enzymes at d 70. The addition of organic Fe-peptide complexes presents greater beneficial effects on enhancing tissue Fe accumulation and Fe-containing enzyme activities, which may be involved in different gene expression patterns related to iron intake and transport in tissues of weaned pigs.

## Introduction

The phenomenon of piglet anemia in production has been paid more and more attention. Piglets are prone to iron deficiency anemia for several reasons, including low iron stores at birth, inadequate sow milk supply, and rapid growth rate (1, 2). For decades, pigs have been selected for large litters, high birth weights and fast growth, which has resulted in greater body blood volume, red blood cells (RBC) count, and iron demands (3). Additionally, weaned piglets have a limited ability to utilize dietary iron (4). Therefore, various iron supplementation strategies have been shown to be beneficial in reducing anemia, as evaluated by measuring the RBC indices and iron status (5). Several attempts have been made to enhance iron status of piglets by iron injection (dosage of 200 mg of iron dextran) or dietary supplementation (100 mg Fe/kg of a corn-soybean meal basal diet as FeSO_4_, Fe-Gly or Heme) without significant effect on growth performance and hematologic variables (6, 7). These results may be attributed to the inability of iron sources and dietary supplemental levels in providing sufficient iron uptake to meet iron requirements. For example, the use of inorganic iron sources is known to affect the absorption and utilization of iron, showing lower bioavailability in animal diets. Organic iron, as an alternative to inorganic iron, presents the advantages of good bioavailability, high absorbability, excellent stability and high safety (8, 9). Among the different types of organic iron supplements, Fe-peptide chelating complexes have been received increasing attention in improving iron bioavailability, with regard to biological effects and intestinal iron absorption properties (10). Organic Fe-peptide chelating complexes bind with iron to form soluble chelate that avoids iron precipitation, and are absorbed through the intestinal peptide absorption pathway, while regulating the proliferation and differentiation of enterocytes through the interaction with the intestinal iron absorption pathway. However, data concerning the effects of Fe-peptide chelating complexes dietary supplementation on iron uptake and transport in piglets are relatively limited. Therefore, the objective of the current study is to evaluate the effects of replacing inorganic Fe with organic Fe-peptide complex on growth performance, hematological parameters, Fe status and activities of enzymes and gene expression related to the iron intake and transport in tissues of weaned pigs.

## Materials and methods

### Animals and diets

The animal care and use protocol was approved by the Animal Care and Use Committee of South China Agricultural University (SCAU-10564), and the study was conducted following the Regulations for the Administration of Affairs Concerning Experimental Animals.

A total of 480 weaned pigs (Duroc X Landrace) at 28 d with an average initial body weight of 8.14 ± 0.71 kg were allotted to four treatments (approximately 0.4 m^2^ of floor space/pig) as a factorial arrangement of treatments, with 30 pigs/pen (male: female = 1:1) and 4 replicate pens/treatment. Four treatments of Fe in the diet were performed: control basal diet (Con, Table 1); Con + 150 mg Fe/kg as inorganic Fe (iFe); Con + 75 mg Fe/kg as inorganic Fe + 75 mg Fe/kg as organic Fe-peptide complex (iFe+oFe), and Con + 150 mg of Fe/kg as organic Fe-peptide complex(oFe). The corn-soybean meal basal diet was formulated to meet nutrient requirements other than iron according to the recommendation of NRC (11). The supplemental Fe level added to the basal diet was considered industry standards, thus exceeding the NRC (11) requirements. Lysine and methionine, as the first and second limited amino acid (AA) in weaned pig diets, could eliminate the effect of additional AA produced by iron-peptide complexes and were considered to AA content in the diets. Therefore, lysine and methionine levels in the control diet or diets supplemented with inorganic iron were balanced by adding synthetic lysine-HCl and DL-methionine depending on the amount of lysine and methionine supplementation from the source of the Fe-peptide complex. Each pen contained two stainless steel nipple drinkers and a four-hole, stainless steel feeder. The ambient temperatures were initially set at 28°C but were adjusted as needed to remain within pigs' comfort zone. Pigs had *ad libitum* access to diets and water during the 42 experimental days from d 28 to d 70. The body weight and feed intake of pigs were measured by pens on d 28 and d 70 of the trial, and then average daily gain (ADG), average daily feed intake (ADFI) and the ratio of feed intake to gain (F/G) were calculated as following.


$$\begin{array}{l} \text{ADG = }(\text{off}-\text{test pen weight }-\text{ allotment pen weight})\text{/}\\ \;(\text{pigs placed}\times \text{d on}-\text{test}) \end{array}$$

**Table 1:** Composition and nutrient levels of the basal diets (as-fed basis).

**Item**	**Post-weaning period (d 28-70)**
**Ingredient, g/kg**	
Corn	391.5
Extruded corn	186.0
Soybean meal	100.0
Fermented soybean meal	50.0
Extruded soybean	100.0
Grease powder	20.0
Whey powder	12.5
Brown sugar	20.0
Grease powder	10.0
Fish meal	12.0
Fish peptide	10.0
Limestone	6.15
Dicalcium phosphate	9.8
Salt	3.5
DL-Met (98%)	1.0
L-Lys sulphate^a^	5.6
Micronutrients^a^	2.0
**Nutrient composition, %**	
DE, MJ/kg	3,450
CP^b^	18.2
Lys	1.35
Met	0.60
Met + Cys	0.90
Ca^b^	0.67
Nonphytate P^b^	0.35
Fe^b^, mg/kg	75.8

a Supplied the following per kilogram of diet: vitamin A, 2200 IU; vitamin D_3_, 220 IU; vitamin E, 16 IU; vitamin K_3_, 0.5 mg; vitamin B_1_,1.5 mg; vitamin B_2_,4.0 mg; vitamin B_6_, 3.0 g; vitamin B_12_, 0.02 mg; pantothenate, 12 mg; nicotinic acid, 30 mg; Fe, 0 mg; Cu, 6 mg; Zn, 100 mg; Mn, 4 mg; Se, 0.3 mg; I, 0.14 mg; colistin, 40 mg; 50% olaquindox, 20 mg.

b Analyzed values based on triplicate determinations.

The feed intake was calculated by subtracting the residual feed from the feed provided, after making corrections for dry matter content of the feed ADFI = (Total feed eaten) / (pigs placed × d on-test). F/G is calculated by dividing the kg of feed eaten daily, by the kg of live weight gained daily.

After 12 h of fasting, the same two piglets per replicate pen were bled *via* cannulation of the ear veins puncture for repeat sampling of small volumes (<4 mL) at birth before dietary iron treatment, and on d 42, 56 and 70, respectively. At d 70, these selected pigs were anesthetized with an intravenous injection of pentobarbital sodium (50 mg/kg, Tc-P8411, Toscience Biotechnology, Shanghai, China) and sacrificed for tissue samples collection. Samples of their liver, kidney, and spleen were collected and a subsample was frozen at −20°C for analyses of iron content and catalase (CAT) and succinate dehydrogenase (SDH) activities. Another subsample of liver and bone marrow tissues were frozen in liquid nitrogen for gene expression assays. At the end of the experiment, the rest of piglets were removed and were fed with a commercial feed to meet the nutrients requirements. Lighting and feeding management were performed according to the instructions of piglets' management guidelines. The recovery performance standards and normal behaviors were done to evaluate the optimum welfare weekly.

### Sample collections and analyses

Blood samples (2 to 3 mL) were placed on ice and transported to the laboratory for hematological measurements. Liver, kidney, and spleen samples were homogenized in ice-cold 10% (wt/vol) physiological saline for 1 min using a tissue grinder (T 18 D S25; IKA Group, Staufen, Germany) and then processed for 1 min using an ultrasonic wave cell grinder (JY92-11; Ningbo Scientz Biotechnology Co., Ltd., Ningbo, Jiangsu, P. R. China) for 1 min (1 s, with 2 s interval). Then, the homogenates were centrifuged at 1,000 × *g* for 15 min at 4°C to harvest the supernatants for immediately analyzing total protein content and CAT and SDH activities.

Iron contents in the diets and tissues were determined by inductively coupled plasma emission spectroscopy after wet digestions with HNO_3_ and HCIO_4_ as described by Zhang et al. (12). The iron analysis was validated with bovine liver powder as a standard reference material (GBW [E] 080193; National Institute of Standards and Technology, Beijing, China). Concentrations of Ca and CP in feed ingredient or diet samples were determined as described by the AOAC (13). Lysine and methionine contents of organic iron were analyzed by an automatic amino acid analyzer L-8800 (Hitachi, Tokyo, Japan). Hemoglobin (HGB) and mean corpuscular hemoglobin concentration (MCHC) levels in blood were determined using an automated AVIDIA 2010 analyzer (Siemens, Germany). CAT activities were analyzed by Goth's colorimetric method (14) with a commercial available test kit (A022-1-1; Jiancheng Bioengineering Institute, Nanjing, China). The activities of SDH were analyzed using a colorimetric method with a commercially available test kit (A002; Jiancheng Bioengineering Institute, Nanjing, China). Total protein concentrations in the supernatants of tissue homogenates were assayed by a BCA Protein Assay kit (23225; Thermo Scientific, Rockford, IL, USA) as per the manufacturer's instruction. The activities of these enzymes were expressed on a per mg protein basis.

Total RNA in the liver and marrow were isolated using TRIZOL reagent (15596018; Life Technologies, Carlsbad, CA, USA) according to the manufacturer's instructions. Reverse transcription was performed using the PrimeScript RT reagent Kit with gDNA Eraser (RR047A; Takara Bio Inc., Otsu, Japan) according to the manufacturer's protocols. The obtained cDNA was used to determine the gene mRNA expression of *Ferritin, transferrin (Tf), transferrin receptor 1 (TfR1), and nuclear receptor coactivator 4 (NCOA4)* by ABI 7500 RT-PCR detection system (Applied Biosystems, Merelbeke, Belgium, USA) using Fast SYBR Green Master Mix. Information of primers was summarized in Table 2. The relative gene expression was quantified by normalizing to the mRNA expression of β*-actin* as per the 2^−Δ*ΔCt*^ method (15).

**Table 2:** The information of PCR primers.

**Genes**	**Primer sequences (5^′^-3^′^)**	**Product size(bp)**	**Accession No**.
*β-actin*	F: CCAGCACCATGAAGATCAAGATC	102	NM_001614.5
	R: ACATCTGCTGGAAGGTGGACA		
*TfR1*	F: GGCTGTATTCTGCTCGTGGA	195	NM_214001.1
	R: AGCCAGAGCCCCAGAAGATA		
*NCOA4*	F: CCTGAGCCTGAGAAACAT	134	NM_001006.5
	R: TAAAGGGACACCACGAAG		
*Ferritin*	F: GCCAAATACTTTCTTCACCA	142	NM_213975.1
	R: CAGTCAGCCCATTCTCCC		
*Tf*	F: GCGGGTTTGGTATTTGAGGC	153	NM_212787.1
	R: GGTTTGTGGATTATCTTTCTGCCC		

Tf, transferrin; TfR1, transferrin receptor 1; NCOA4, nuclear receptor coactivator 4.

### Statistical analyses

Data were analyzed by one-way ANOVA using the PROC GLM procedure of the SAS (SAS Inst. Inc., Cary, NC) and all data are presented as mean ± SD. The number of replicate served as the experimental unit. Differences among means were tested by the Fisher's Least Significance Difference test method, and statistical significance was set at *P* ≤ 0.05.

## Results

### Growth performance and blood hemoglobin and MCHC levels

No significant differences (*P* > 0.05) were observed between pigs fed the control diet and those fed the iron-supplemented diets in terms of final body weight, ADG, ADFI, and F/G (Table 3). There were also no significant differences (*P* > 0.05) in hemoglobin and MCHC contents at all sampling time points between the pigs fed the control diet and those fed Fe-supplemented diets (Table 4).

**Table 3:** Effects of different iron sources on growth performance of weaned piglets from d 28 to d 70.

**Items**	**Dietary Fe sources**				***P*-value**
	**Con**	**iFe**	**iFe**+ **oFe**	**oFe**	
Final weight, kg	26.5 ± 1.68	26.7 ± 1.55	25.9 ± 2.31	26.2 ± 2.38	0.74
Average daily gain, g/d	430.5 ± 45.3	460.7 ± 35.1	443.9 ± 50.3	421.6 ± 50.2	0.71
Average feed intake, g/d	833.3 ± 82.9	850.9 ± 45.6	816.4 ± 64.4	888.3 ± 77.0	0.56
Feed/gain	1.90 ± 0.11	1.89 ± 0.14	1.86 ± 0.12	1.95 ± 0.11	0.82

All data were presented as mean ± SD. Mean represented the average value of 4 replicates (n = 4). Con, control basal diet; iFe, Con + 150 mg Fe/kg as inorganic Fe; iFe+ oFe, Con + 75 mg Fe/kg as inorganic Fe + 75 mg Fe/kg as organic Fe-peptide complex; oFe, Con + 150 mg of Fe/kg as organic Fe-peptide complex.

**Table 4:** Effects of different dietary iron sources on hemoglobin and MCHC levels of weaned piglets from d 28 to d 70.

**Items**	**Samlping day**	**Dietary Fe sources**				***P*-value**
		**Con**	**iFe**	**iFe**+ **oFe**	**oFe**	
Hemoglobin, g/L	d 28	115.3 ± 9.4	116.3 ± 6.3	113.3 ± 10.4	112.0 ± 9.6	0.93
	d 42	97.5 ± 9.3	109.0 ± 6.7	103.0 ± 8.2	113.0 ± 3.7	0.38
	d 57	98.5 ± 9.0	112.0 ± 10.2	101.8 ± 8.6	96.8 ± 4.1	0.41
	d 70	95.5± 11.0	99.3± 8.4	100.5 ± 9.6	97.5 ± 4.0	0.93
	d 28	446.2± 56.7	404.6 ± 69.4	438.4 ± 87.9	433.6 ± 67.2	0.69
MCHC, g/L	d 42	353.9 ± 28.0	395.7 ± 38.5	373.9 ± 37.0	410.2 ± 36.1	0.38
	d 56	215.4 ± 28.8	251.3 ± 17.2	218.3 ± 15.5	217.1 ± 11.9	0.28
	d 70	310.2 ± 67.2	322.4 ± 36.1	326.5 ± 23.4	316.7 ± 26.9	0.93

All data were presented as mean ± SD. Mean represented the average value of 4 replicates (n = 4). Con, control basal diet; iFe, Con + 150 mg Fe/kg as inorganic Fe; iFe+oFe, Con + 75 mg Fe/kg as inorganic Fe + 75 mg Fe/kg as organic Fe-peptide complex; oFe, Con + 150 mg of Fe/kg as organic Fe-peptide complex.

### Tissue iron content and activities of fe-containing enzymes

Compared with the control piglets, piglets in Fe supplemented groups had greater iron concentrations in the liver, kidney and spleen regardless of the iron source (*P* < 0.05; Table 5), while piglets in oFe group had greater tissue iron concentrations than iFe or iFe+oFe groups (*P* < 0.05). Piglets in either iFe+oFe or oFe groups showed greater CAT and SDH activities in liver than Con group (*P* < 0.05; Table 5), while piglets in oFe group had greater CAT and SDH activities in liver than those in iFe group (*P* < 0.05). There were no significant differences between Con and iFe groups as well as between iFe+oFe and oFe groups. CAT and SDH activities were higher in the oFe than in the Con group in kidney of piglet (*P* < 0.05), but there were no significant differences between the other groups. There was no difference in the activities of CAT and SDH in spleen among different groups.

**Table 5:** Effects of different dietary iron sources on Fe contents and Fe-containing enzyme activities in tissues of weaned piglets at d 70.

**Tissues**	**Indices**	**Dietary Fe sources**				***P*-value**
		**Con**	**iFe**	**iFe**+ **oFe**	**oFe**	
Liver	Fe content, mg/kg	147.8 ± 23.4^d^	238.7 ± 13.8^c^	285.8 ± 19.9^b^	398.6 ± 12.4^a^	<0.0001
	CAT, U/ mg protein	74.8 ± 3.2^c^	75.6 ± 2.1^bc^	82.4 ± 2.7^ab^	89.0 ± 4.7^a^	<0.001
	SDH, U/ mg protein	82.8 ± 1.3^c^	86.2 ± 1.9^bc^	94.0 ± 2.8^ab^	99.9 ± 4.6^a^	0.004
Kidney	Fe content, mg/kg	90.3 ± 5.3^d^	110.4 ± 7.4^c^	118.8 ± 4.6^b^	127.6 ± 3.1^a^	<0.0001
	CAT, U/ mg protein	34.5 ± 5.1^b^	36.3 ± 5.5^ab^	37.2 ± 6.8^ab^	43.3 ± 1.1^a^	0.04
	SDH, U/ mg protein	56.3 ± 3.6^b^	59.7 ± 3.2^ab^	65.9 ± 5.2^ab^	69.7 ± 1.3^a^	0.005
Spleen	Fe content, mg/kg	60.1 ± 3.6^d^	76.0 ± 2.5^c^	94.2 ± 3.9^b^	130.8 ± 2.0^a^	<0.0001
	CAT, U/ mg protein	16.4 ± 3.3	12.4 ± 1.6	15.3 ± 2.8	12.7 ± 2.4	0.14
	SDH, U/ mg protein	46.8 ± 11.7	49.7 ± 9.2	53.9 ± 7.9	46.4 ± 9.5	0.42

All data were presented as mean ± SD. Mean represented the average value of 4 replicates (n = 4). Lacking common letters (a–c) significant differences at P <0.05. Con, control basal diet; iFe, Con + 150 mg Fe/kg as inorganic Fe; iFe+oFe, Con + 75 mg Fe/kg as inorganic Fe + 75 mg Fe/kg as organic Fe-peptide complex; oFe, Con + 150 mg of Fe/kg as organic Fe-peptide complex.

### Gene expression related to iron homeostasis

Dietary iron supplementation increased (*P* < 0.05) the expression of *NCOA4* mRNA and decreased (*P* < 0.05) the expression of *TfR1* mRNA expression in liver. However, there were no significant differences (*P* > 0.05) in above gene mRNA expressions in the liver of piglets fed the diets with different iron sources. The *TfR1, NCOA4* and *Ferritin* mRNA expressions of bone marrow were greater (*P* < 0.05) than that of the control and oFe groups. There were no significant differences (*P* > 0.05) in the above gene mRNA expressions of bone marrow between the piglets fed the control diet and those fed oFe-supplemented diets.

## Discussion

In the present study, no significant effects were observed for growth performance in piglets from d 28 to d 70 after dietary 150 mg Fe/kg supplementation originated by different sources in the corn-soybean meal basal diet. On the one hand, both hepatic iron reserves and the sow's milk are sufficient to maintain growth after weaning. On the other hand, dietary supplementation with no supplementation of iron could provide sufficient iron uptake to meet the iron requirements. As reported previously, the corn-soybean meal basal diet supplemented with 100 mg Fe/kg as FeSO_4_, Fe-Gly or Heme did not affect the growth performance of weanling pigs aged from d 25 to d 53 (6). However, the inconsistent result showed that the iron-deficient diet (25.8 mg/kg) supplemented with 100 mg/kg ferrous glycine significantly improved growth performance in weanling pigs during the 21-day trial period (16). The discrepancy between the studies may depend on the differences in the supplemental Fe levels, Fe sources, Fe content in basal diets and Fe depletion periods of the piglets. Recently, larger and faster growing pigs have shown signs of anemia earlier because of their increasing tissue growth and the corresponding need for a greater blood volume (3). Therefore, the aged effect of iron sources in hematological parameters was determined in the present study. Several studies have focused on the level of hemoglobin and the cut-off values for anemia in piglets (17, 18). Supplementation of purified or semi-purified diets with different iron contents and sources (~20.2 mg Fe/kg), below requirements, resulted in higher concentrations of hemoglobin and hematocrit in piglets (19) and chick broilers (20). In the present study, there were no significant differences in hematological indices of hemoglobin and MCHC levels between the control diet and iron-supplemented diets during the experimental period, suggesting that hematological indices of piglets may not be sufficient sensitivity required to detect differences of iron status in piglets when a commercial corn-soybean meal diet containing a higher iron content of 75.8 mg Fe/kg is adopted, which are consistent with the results of the previous studies in piglets (7, 17, 21) and broilers (12). Iron depletion using a practical basal diet for 36 days after weaning may be not enough to present a subclinical form of iron deficiency in micro erythrocytes.

Target tissue accumulations of iron have always been considered to be sensitive criteria to assess the iron status and relative bioavailability of iron sources (12, 22). In the present study, supplementation with 150 mg Fe/kg, irrespective of the source of iron deficiency diet, significantly increased iron content in liver, kidney and spleen. In particular, iron retention in tissue increased as the organic iron peptide complexes replaced inorganic iron levels. Iron amino acid complex (23) and iron glycine chelate (24) were more effective in improving iron status of weanling pigs than iron sulfate. Collectively, these results imply that organic Fe-peptide complex may have better bioavailability than inorganic iron salts fed to lactating pigs, due to its high absorption and good stability. Firstly, Organic Fe-peptide complexes are more stable in the digestive tract and less prone to interactions and antagonisms due to binding to organic molecules, and as a result, improve iron efficiency by intestinal iron absorption pathway (25). Zhang et al. (12) and Cao et al. (22) indicated that the relative bioavailability of organic iron sources was closely related to their chelation strength, and organic iron sources with greater chelation strengths showed higher iron bioavailability. Secondly, Fe-peptide complexes have been reported to be absorbed by intestinal peptide absorption pathway (24). Iron is an essential element required for the functions of numerous enzymes, such as CAT, SDH, and so on (26). The activities of enzymes are affected by Fe level (27), and they are used as indices to evaluate iron supplementation efficiency in pigs. In the present experiment, compared with control group, CAT activities in the liver and kidney of pigs increased with dietary Fe supplementation regardless of Fe sources, and the same tendency of SDH activity in liver was observed in broilers (12) and piglets. In addition, piglets fed with oFe diet had greater CAT and SDH activities in kidney than those fed Con diet, with no significant differences observed among other groups. These results indicate the sensitivity of Fe status and the activities of Fe-containing enzymes in tissues over indicators of growth performance and mature erythrocytes in detecting the bioavailability of iron sources in weaned piglets.

Iron uptake, transport and retention are indicators of the efficiency of absorption and bioavailability of supplemental Fe level and sources (4). Hepatic iron stores represent the primary source of iron in response to the metabolic demands of the organism. Ferritin, a ubiquitous and highly conserved iron-chelating protein, is considered as the major iron storage protein that maintains a large iron core in its cavity and has ferroxidase activity (28). However, no significant effect of on hepatic *ferritin* mRNA expression was observed in pigs between the control and Fe-supplemented groups in the current study, while *ferritin* mRNA expression in bone marrow was increased in either iFe or iFe+oFe group compared with the control and oFe groups (Table 6). This is because transferrin is saturated, and the highly available free iron form induces ferritin expression. It was speculated that low levels of free iron or high levels of chelated iron were present in bone marrow of piglets fed the control and oFe diets, respectively. In addition, delivery of ferritin to lysosomes required NCOA4 protein to enable cells to use stored iron, which recruits ferritin as a cargo molecule (29). Our results show that there is a decrease in the *NCOA4* mRNA expression in liver and marrow under iron depletion. *In vitro* study revealed that *NCOA4*-deficient cells were unable to degrade ferritin, leading to a decrease in bioavailable intracellular iron (30). However, *NCOA4* mRNA expression was observed to be significantly decreased in the bone marrow of piglet fed oFe diet. This finding could be possibly attributed to the degradation of soluble NCOA4 and ferritin by the macro autophagy pathway in an effort to prevent relative excessive iron storage under prolonged iron repletion by Fe-peptide complex with high bioavailability. Our results suggest that the NCOA4-ferritin axis may be involved in modulating intracellular iron homeostasis in accordance with cellular iron availability. Under physiological circumstances, the major iron uptake route utilized by most cells involves Tf-bound iron, which is bound to TfR1 and then internalized through receptor-mediated endocytosis (31). In the present study, dietary iron supplementation regardless of sources failed to affect *Tf* mRNA expression in liver and marrow. Translation of *TfR1* mRNA in both liver and marrow was increased under iron deficient conditions, which was consistent with the increased tissue Fe accumulation due to the resultant reduction in cellular iron export. The transferring receptor serves as a sensitive indicator of iron deficiency and can be used to provide a reliable index of iron stores.

**Table 6:** Effects of different dietary iron sources on gene expression related to related to iron homeostasis in tissues of weaned piglets at d 70.

**Tissues**	**Gene**	**Dietary Fe sources**				**P-value**
		**Con**	**iFe**	**iFe**+ **oFe**	**oFe**	
Liver	*Tf*	1.52 ± 0.62	1.14 ± 0.52	1.66 ± 0.70	1.86 ± 0.64	0.51
	*TfR1*	3.39 ± 0.92^a^	1.67 ± 0.96^b^	1.42 ± 0.40^b^	1.41 ± 0.38^b^	0.02
	*NCOA4*	0.87 ± 0.24^b^	2.03 ± 0.92^a^	2.26 ± 1.24^a^	2.60 ± 0.65^a^	0.04
	*Ferritin*	1.94 ± 0.28	1.67 ± 0.74	1.29 ± 0.59	1.47 ± 0.28	0.61
Bone marrow	*Tf*	1.32 ± 0.70	1.14 ± 0.77	1.26 ± 0.66	1.46 ± 0.87	0.67
	*TfR1*	0.41 ± 0.25^c^	7.14 ± 1.60^a^	3.48 ± 1.33^a^	1.70 ± 0.68^b^	0.002
	*NCOA4*	0.61 ± 0.19^b^	5.74 ± 1.26^a^	4.20 ± 1.04^a^	0.75 ± 0.46^b^	0.03
	*Ferritin*	0.87 ± 0.28^b^	2.51 ± 0.66^a^	3.68 ± 1.27^a^	1.14 ± 0.18^b^	0.03

All data were presented as mean ± SD. Mean represented the average value of 4 replicates (n = 4). Lacking common letters (a-c) significant differences at P <0.05. The GAPDH expression was used to normalize the expressions of the targeted genes. Con, control basal diet; iFe, Con + 150 mg Fe/kg as inorganic Fe; iFe+oFe, Con + 75 mg Fe/kg as inorganic Fe + 75 mg Fe/kg as organic Fe-peptide complex; oFe, Con + 150 mg of Fe/kg as organic Fe-peptide complex; Tf, transferrin; TfR1, transferrin receptor 1; NCOA4, nuclear receptor coactivator 4.

## Conclusion

In conclusion, the levels and sources of dietary iron supplementation do not influence growth performance and hematological parameters in weaned piglets fed a corn-soybean meal basal diet supplemented with 150 mg Fe/kg from d 28 to d 70. Supplementation of 150 mg Fe/kg in the basal diet regardless of sources increases tissue iron status and activities of Fe-containing enzymes and the addition of organic Fe-peptide complex presents greater beneficial effects on iron bioavailability of weaned pigs at d 70. In addition, the iron homeostasis regulated by iron sources may involve different gene expression patterns related to iron intake and transport in tissues.

## Data availability statement

The original contributions presented in the study are included in the article/supplementary material, further inquiries can be directed to the corresponding authors.

## Ethics statement

The animal study was reviewed and approved by the Animal Care and Use Committee of South China Agricultural University (SCAU-10564) and the study was conducted following the Regulations for the Administration of Affairs Concerning Experimental Animals.

## Author contributions

All authors listed have made a substantial, direct, and intellectual contribution to the work and approved it for publication.
